# Mapping Hot Spots and Global Research Trends in Exergaming Between 1997 and 2024: Bibliometric Analysis

**DOI:** 10.2196/66738

**Published:** 2025-08-25

**Authors:** Abdullah Kayhan, Elif Kose, Burcu Kayhan, Nan Zeng

**Affiliations:** 1 Department of Recreation, Faculty of Sport Sciences, Akdeniz University, Antalya, Turkey; 2 Department of Recreation, Institute of Social Sciences, Akdeniz University, Antalya, Turkey; 3 Department of Exercise and Health Sciences, Manning College of Nursing and Health Sciences, University of Massachusetts Boston, 100 Morrissey Boulevard, Boston, MA, 02125, United States, 1 617-287-4867

**Keywords:** exergaming, active video games, physical activity, health outcomes, bibliometric analysis

## Abstract

**Background:**

Exergaming, the combination of exercise and gaming, has emerged as an important area in physical activity (PA) research. By leveraging advances in video game technology, exergaming supports both physical and mental health. This growing interest in exergaming has increasingly attracted researchers over the years. Examining the development of exergaming research with a bibliometric approach is thought to offer valuable perspectives to researchers by revealing the trends and main contributions in the field.

**Objective:**

This study aims to identify the most researched concepts and topics in the field of exergaming; track the changes of trending topics over the years; identify the most influential journals as well as the authors who have contributed the most to the field; identify groundbreaking studies and neglected topics that shape future work; and reveal the countries, institutions, and collaborations that have contributed the most to the field. It also aims to identify research gaps in the field of exergaming and provide important recommendations for future research.

**Methods:**

A bibliometric analysis covering studies between 1997 and 2024 was conducted using the Web of Science database. The R-based Bibliometrix package and the Biblioshiny web interface were used for data analysis and visualization. The analysis included original research papers and reviews. These analyses provided insights into research trends, citation metrics, and thematic developments.

**Results:**

A total of 1626 studies were analyzed, and the results indicated a steep rise in exergaming research since 2015, peaking in the years 2020‐2021. Major high-impact journals publishing in this area include *Games for Health Journal* and *International Journal of Environmental Research and Public Health*. Researchers who have contributed significantly and enriched the knowledge base of the exergaming field included Gao Zan, Eling de Bruin, and Zeng Nan. The most cited studies were classified into 2 different clusters, namely, cluster 1 that focuses on the concepts of PA, exercise, energy expenditure, and children, while cluster 2 focuses on rehabilitation, balance, adults, and aging. Medicine, information technology, and intention are some of the emerging themes. From a research productivity perspective, there is an undisputed front-runner, the United States, but substantial contributions have definitely come from either the Swiss Federal Institute of Technology or the Karolinska Institute.

**Conclusions:**

Despite significant growth in exergaming research over the last decade, research gaps remain, particularly in understanding how exergaming can be effectively integrated into long-term PA promotion and broader health outcomes. These gaps were identified by the absence or low representation of relevant keywords (eg, “cost-effectiveness,” “community-based intervention,” and “long-term health outcomes”) in thematic mapping and keyword trend analyses and limited citation density in these areas. Future work should explore these issues more systematically to advance the field.

## Introduction

Physical inactivity is a significant risk factor for noncommunicable diseases worldwide [1]. Exergaming plays an important role in preventing these diseases associated with physical inactivity and promoting healthy habits [2]. “Exergaming,” also known as active video games, are video games that incorporate physical activity (PA) into the gaming concept, requiring players to move physically [3].

Leisure-time PA can be influenced by social and environmental factors (lack of time, lack of willpower, lack of friends, unsafe living environments, adverse weather conditions, socioeconomic status, health status, physical disability, etc) [4]. In this regard, exergaming can provide indoor or home-based alternatives that make PA more enjoyable and fun, thereby increasing exercise motivation [5]. Furthermore, unlike traditional sedentary video games, exergaming provides players with an optimal level of PA and double the amount of light to moderate energy expenditure compared to sedentary video gamers [6]. Therefore, exergaming has been used to promote PA and health in healthy adults [7], different age groups [8], and those with pathological conditions [9].

Exergaming increases energy expenditure and PA, promoting an active lifestyle [10]. Exergaming helps with weight management [11] and improving mental health [12] and can prevent sedentary behaviors [13]. The American College of Sports Medicine describes exergaming as “the future of fitness,” and numerous studies highlight its potential to increase PA, especially among children and adolescents [14]. Furthermore, exergaming offers innovative solutions for health and rehabilitation services [15]. A recent systematic review suggested that exergaming may be recommended for patients undergoing cancer treatment [16]. Furthermore, exergaming has been shown to be effective in the rehabilitation of Parkinson disease [17] and in reducing cancer-related fatigue in children undergoing chemotherapy for leukemia [18]. Studies evaluating older adults have shown that exergaming improves various functions such as mental health, cognitive motor skills, and balance [19]. Additionally, exergaming supports psychological health by making PA more enjoyable [20].

Although a growing number of systematic reviews and meta-analyses have examined the effects of exergaming on specific outcomes such as PA promotion in children [11], older adults or clinical populations [16], cognitive function [19], balance improvement [21], and motivation [12,22], these studies primarily provide syntheses of intervention effects or health-related outcomes. However, they do not provide an overview of the evolution, structure, and dynamics of scientific output in this field over time. In contrast, bibliometric analysis sheds light on the historical development of a research field, allowing the identification of scientific production, trends, gaps, and future directions [23,24]. It also enables researchers to assess patterns of authorship, collaboration networks, and thematic trends [25].

Despite the growing popularity of exergaming, bibliometric studies specifically mapping this field remain limited. The few existing studies have largely focused on adjacent or overlapping fields such as PA, virtual reality (VR), augmented reality (AR), or rehabilitation research [26,27]. However, exergaming encompasses a wider range of technologies and applications, including motion-sensing consoles, mobile apps, and digital fitness platforms, which have not been adequately addressed in these previous reviews. Therefore, bibliometric analysis focusing specifically on exergaming is necessary to better understand its thematic breadth, interdisciplinary contributions, and underexplored areas. This study aims to address this gap by providing a scholarly map of the exergaming literature from 1997 to 2024, offering valuable insights for researchers and practitioners interested in advancing this burgeoning field.

In line with the research objective, we have formulated our research questions (RQs) as follows:

- RQ1: What is the distribution of studies published on exergaming by year?
- RQ2: What are the most prolific journals in the field of exergaming?
- RQ3: How is the distribution of the number of publications of the authors who have done the most work in the field of exergaming according to years?
- RQ4: What are the most cited studies and trending topics in the field of exergaming?
- RQ5: What is the subject clustering of studies in the field of exergaming?
- RQ6: What are the most productive countries, institutions, authors, and their collaborations?
- RQ7: What are the past, current, and emerging trends, key contributors, and collaborative networks shaping the field of exergaming research?

## Methods

### Analysis Tool and Database

The bibliometric analysis was conducted in the open-access R-Based Bibliometrix package designed by Aria and Cuccurullo [28]. This has been favored due to its smooth interaction with Web of Science (WoS) and fully exploits the Keyword Plus property for a beautiful analysis of exergaming research [29].

Combining data from different databases can lead to inconsistencies in citation metrics, metadata, and affiliation information, which may negatively impact the accuracy and reliability of the analysis [30]. For this reason, in this research, we decided to use 1 database for the study, that is, the WoS. WoS offers comprehensive options for analysis due to its good citation metrics, elaborate metadata structure, and important tools like Keyword Plus, which provides an increased number of terminologies in addition to keywords provided by the authors [31].

### Search Strategy

The searches included peer-reviewed original and review papers published from 1997 to 2024. According to the search string applied, the earliest accessible indexed record from WoS dated back to 1997. Therefore, 1997 was used as the initial cutoff point for inclusion. Two independent researchers (AK and EK) carried out the searches, and the retrieved data were evaluated independently on a Microsoft Excel database for relevance to the study scope. Search strategies were based on a literature review, and the search prompt was created with search strategies, such as (TI=(“exergame*” OR “gamercising” OR “exergaming” OR “gamercize*” OR “active video game*”)). The detailed search strategy appears in Multimedia Appendix 1. The WoS search resulted in 2663 studies. No language restrictions were applied.

As part of the data cleaning process, duplicate records were removed (n=2), and author names, affiliations, and journal titles were manually reviewed and standardized by 2 researchers to ensure consistency and reduce misclassification. Studies without accessible abstracts or full texts, those published from 2025 onward (n=5), and gray literature (n=1030) were excluded. Gray literature refers to academic work available in journals, books, print, or electronic formats that may be used commercially and are not controlled by publishing entities [32]. Ultimately, 1626 records were included in the analysis.

### Data Analysis and Visualization

Studies meeting the data processing criteria were imported into the user-friendly Biblioshiny interface via R Studio. The analysis evaluated the impact of sources by examining paper counts, h-indexes, total citation (TC) counts, and citations per publication. The h-index is a key metric for assessing the impact of an author or journal [33]. Citations play a critical role in measuring the influence of sources within a research field. Network metrics, such as modularity (Q) and network density, were also used to evaluate the frequency of relationships and clustering structures. A modularity value between 0.4 and 0.8 indicates a well-structured clustering network [34]. Finally, social network analysis was used to visualize the actors and relationships in exergaming research.

### Ethical Considerations

Ethics approval was not required for this study, as human participants were not involved.

## Results

### Descriptive Statistics in the Field of Exergaming

**Figure 1.**
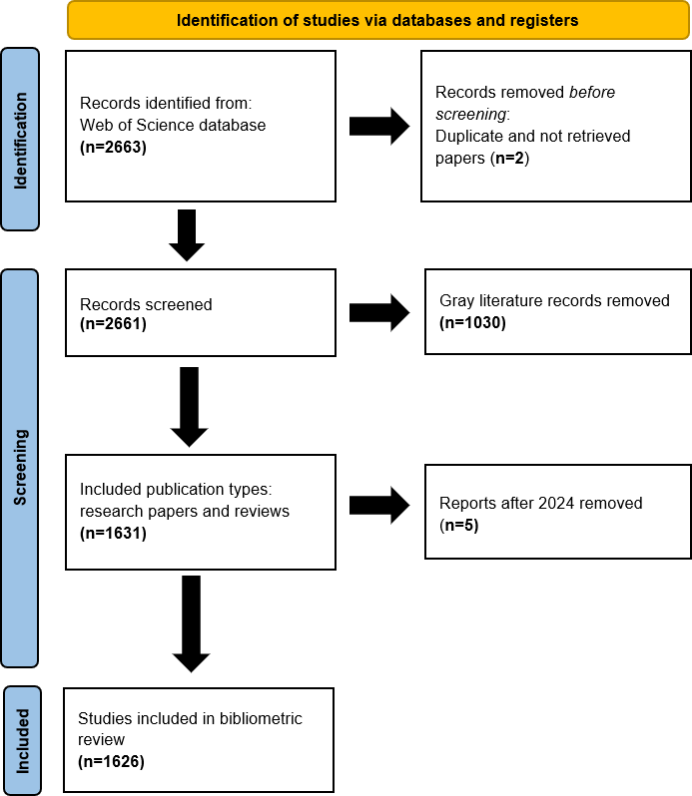
The flowchart for data collection.

The data for the 1626 included studies are presented in Multimedia Appendix 2 and are outlined in the data collection flow diagram (Figure 1).

Table 1 provides information on the key descriptive statistics regarding studies conducted in the exergaming field. The research in this area comprises 1626 documents sourced from 553 different outlets between 1997 and 2024. The annual growth rate of publications is 22.07%, with an average document age of 5.7 years. Notably, each study has received an impressive average citation rate of 21.95%. While there are 38 single-authored documents, 6556 authors have contributed to the field, predominantly through papers (n=1402) and reviews (n=224). The rate of international author collaboration is 26.88%, with an average of 5.57 authors per document. Additionally, 2917 keywords provided by the authors enhance the richness of the dataset. Finally, the 1626 documents have been cited 45,050 times in studies indexed on WoS.

**Table 1.** Descriptive statistics of exergaming studies.

Information	Values
Timespan (years)	1997‐2024
Sources (frequency distribution), n	553
Documents, n	1626
Annual growth rate (%)	22.07
Authors of multiauthored documents, n (%)	6556 (99.4)
Authors of single-authored documents, n (%)	38 (0.6)
International coauthorship (%)	26.88
Coauthors per document (average), n	5.57
Author’s keywords, n	2917
References, n	45,050
Document age (average), n	5.7
Citations per document (average), n	21.95

### Annual Scientific Production and Average Citations Per Year

**Figure 2.**
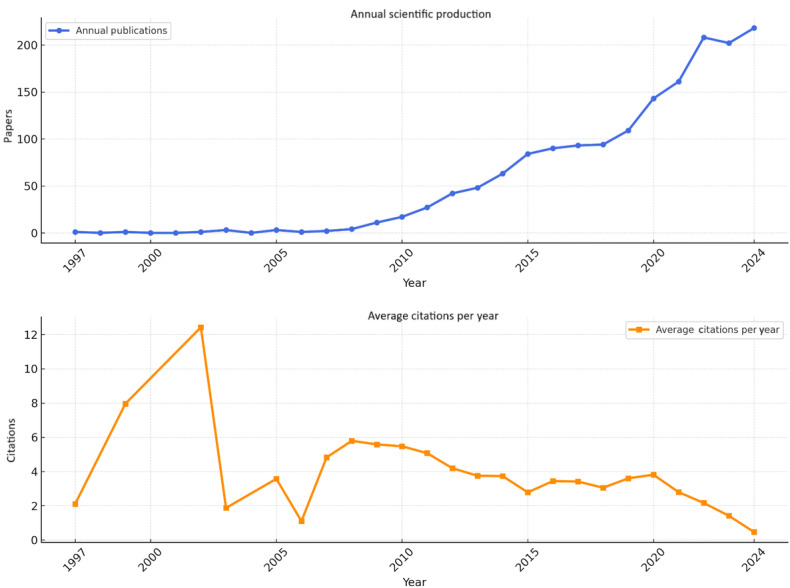
Evaluation of exergaming studies by years and average citations per year.

Figure 2 shows that studies on exergaming began at very low levels in 1997, experienced significant growth starting in 2015, and peaked in 2021. Although there was a slight decline in 2022, scientific output continued in 2023 and 2024. It has been observed that the studies conducted in the field of exergaming and the average citation counts to these studies are not parallel, and there is a decrease in the average citation counts, unlike the number of publications.

### The Most Effective Journals in the Field of Exergaming and Bradford’s Law

An examination of the most relevant journals in the field of exergaming reveals that *Games for Health Journal* ranks first with 139 papers (Tables2  3). This journal is followed by *JMIR Serious Games* with 63 papers. When evaluating the journals with the highest number of citations, *Games for Health Journal* once again tops the list with 1885 citations, followed by *Medicine & Science in Sports & Exercise* with 1241 citations. The local impact and impact factors of the top journals on exergaming are presented in Multimedia Appendix 3. According to the findings of Bradford’s law, *Games for Health Journal* occupies a larger share among the core sources compared to other journals and is positioned within the first core group of journals. It is followed by *JMIR Serious Games* and the *International Journal of Environmental Research and Public Health*. The findings for Bradford’s law are presented in Multimedia Appendix 4.

**Table 2.** Most relevant journals.

Most relevant journals	Papers, n
*Games for Health Journal*	139
*JMIR Serious Games*	63
*International Journal of Environmental Research and Public Health*	54
*PLOS One*	32
*Journal of Clinical Medicine*	28
*Journal of NeuroEngineering and Rehabilitation*	23
*Frontiers in Psychology*	22
*Journal of Medical Internet Research*	22
*Frontiers in Aging Neuroscience*	21
*Applied Sciences—Basel*	19

**Table 3.** Most cited journals.

Most cited journals	Citation counts, n
*Games for Health Journal*	1885
*Medicine and Science in Sports and Exercise*	1241
*PLOS One*	929
*Archives of Physical Medicine and Rehabilitation*	882
*Journal of NeuroEngineering and Rehabilitation*	702
*International Journal of Environmental Research and Public Health*	693
*Physical Therapy & Rehabilitation Journal*	600
*Computers in Human Behavior*	558
*Journal of Physical Activity and Health*	548
*Journal of Sport and Health Science*	541

### Authors’ Productivity and the Distribution of Their Productivity Over the Years

**Figure 3.**
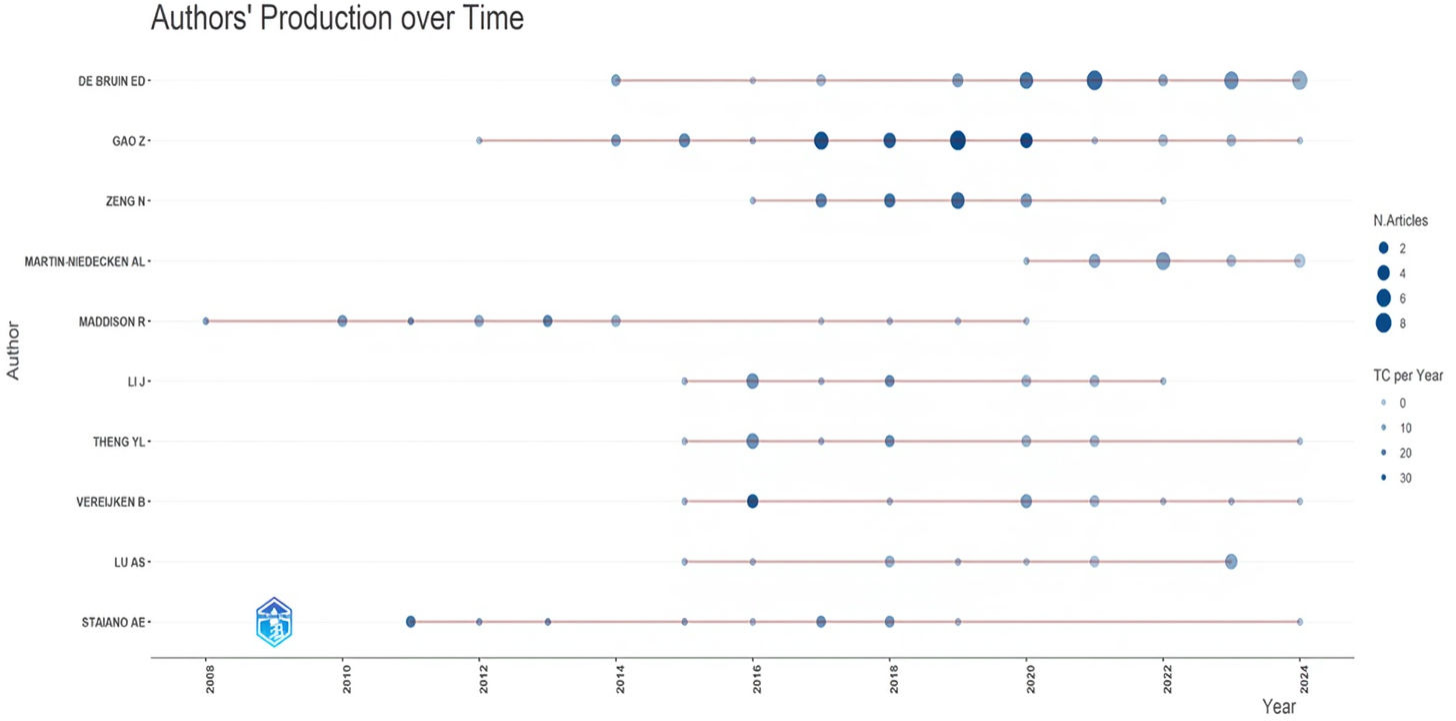
Productivity of authors working on exergaming by year. TC: total citation.

Figure 3 shows the publication performance of the most productive authors on exergaming over the years. Gao Zan (n=36) and Eling de Bruin (n=36) are the most productive authors in this field and continue to work on related studies. The analysis of author productivity based on Lotka’s law reveals that 79.74% (n=5226) of exergaming researchers have contributed with only 1 publication, 12.85% (n=842) have contributed with 2 publications, and 3.74% (n=245) with 3 publications. These findings do not fully align with Lotka’s law. The findings on Lotka’s law are presented in Multimedia Appendix 5.

### Most Cited Sources

Table 4 shows the most cited papers on exergaming, demonstrating the impact and importance of research in this area. The most cited paper (TC=386) by Biddiss and Irwin [35]. The second most cited paper (TC=345) by Ijaz et al [5].

**Table 4.** Globally most cited documents on exergaming.

Title	Author and source	TC^a^	TCPY^b^	NTC^c^
1. Active video games to promote physical activity in children and youth: a systematic review	Biddiss and Irwin [Bibr R33][35]	386	24.13	4.41
2. Player Experience of Needs Satisfaction (PENS) in an immersive virtual reality exercise platform describes motivation and enjoyment	Ijaz et al [Bibr R5][5]	345	57.50	15.08
3. Exergaming and older adult cognition: a cluster randomized clinical trial	Anderson-Hanley et al [36]	304	21.71	5.18
4. Option exercise games: an application to the equilibrium investment strategies of firms	Grenadier [37]	298	12.42	1.00
5. Is playing exergames really exercising? A meta-analysis of energy expenditure in active video games	Peng et al [10]	295	19.67	3.88
6. Effects of interactive physical-activity video-game training on physical and cognitive function in older adults	Maillot et al [38]	289	20.64	4.92
7. Exergames for physical education courses: physical, social, and cognitive benefits	Staiano and Calvert [39]	273	18.20	3.59
8. Need satisfaction supportive game features as motivational determinants: an experimental study of a self-determination theory guided exergame	Peng et al [40]	250	17.86	4.26
9. Playing active video games increases energy expenditure in children	Graf et al [41]	250	14.71	2.63
10. Efficacy and safety of non-immersive virtual reality exercising in stroke rehabilitation (EVREST): a randomised, multicentre, single-blind, controlled trial	Saposnik et al [42]	248	24.80	7.21

a TC: total citation.

b TCPY: total citation per year.

c NTC: normalized total citation.

### Trending Topics

**Figure 4.**
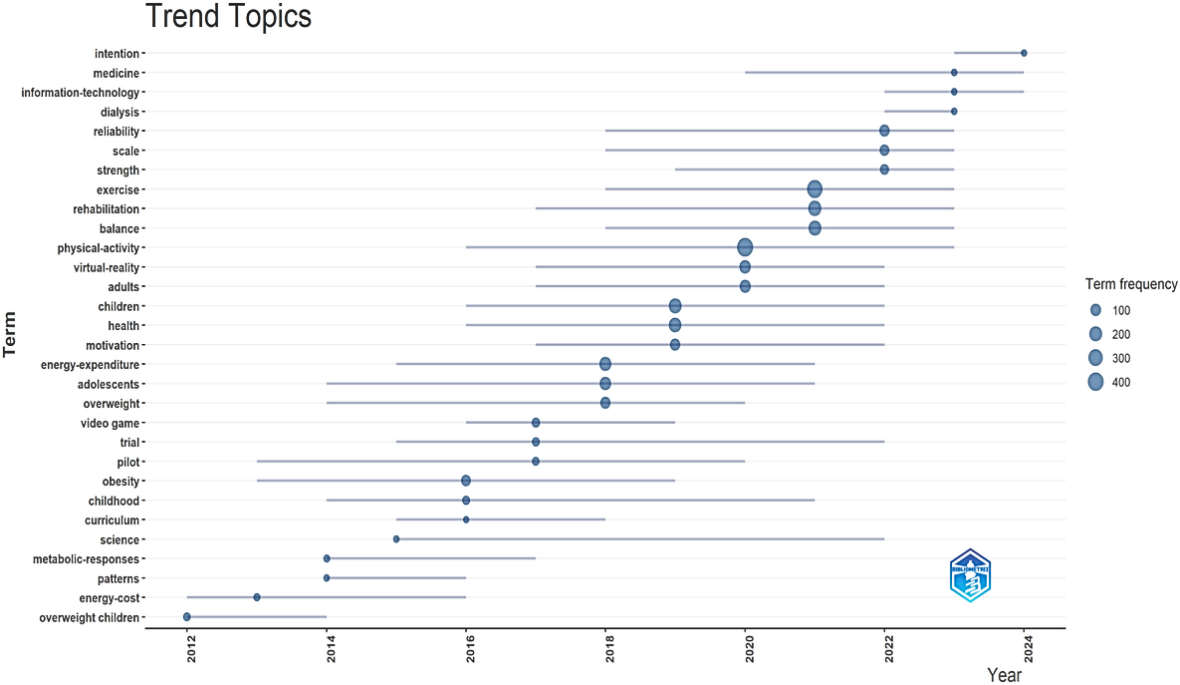
Popular exergaming topics by year.

Figure 4 presents the trending topics in exergaming research over time, showing how these topics have changed over the years. Topics such as “health,” “physical activity,” “exercise,” “virtual reality,” and “obesity” have typically been focal points in this field. In recent years, topics such as “medicine,” “information technology,” and “intention” have emerged.

### Thematic Mapping

**Figure 5.**
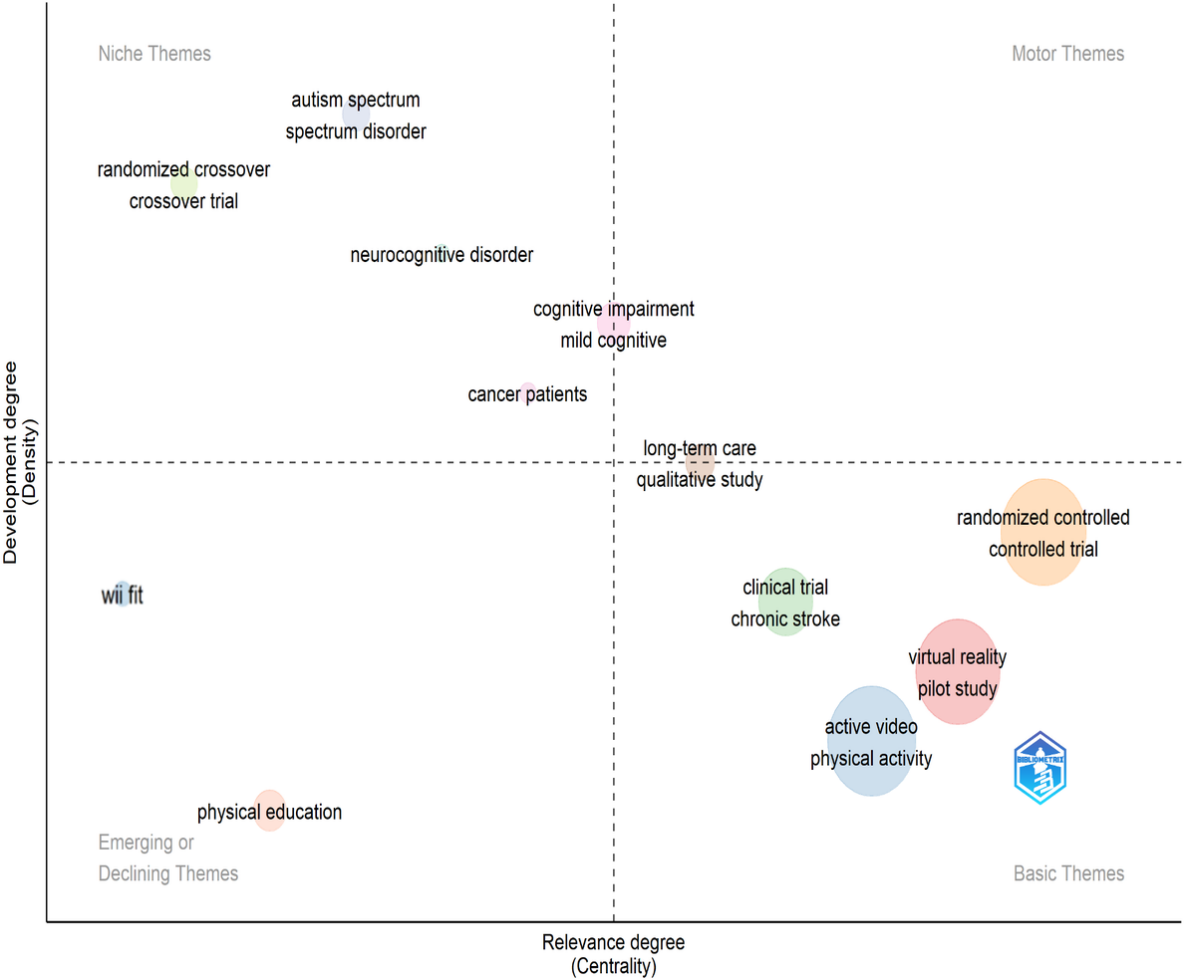
Thematic map findings for exergaming research.

Figure 5 illustrates the development and centrality degrees of exergaming research themes. Motor themes represent well-developed and highly relevant core topics in exergaming research. No specific terms were identified in this region.

Basic themes encompass topics with high centrality but low density. These themes are foundational to research and represent broadly discussed areas. Notable terms in this region include “randomized controlled trial,” “virtual reality,” “physical activity,” “active video,” and “pilot study.”

Niche themes refer to topics with low centrality but high density. These themes represent specific research topics and specialized subfields within the domain. Terms such as “autism spectrum,” “randomized crossover,” and “neurocognitive disorder” are prominent in this region.

Emerging or declining themes are characterized by low centrality and low density. These themes represent either emerging or diminishing areas of interest in exergaming research. Notable terms in this region include “Wii fit” and “physical education.”

### Collaboration

**Figure 6.**
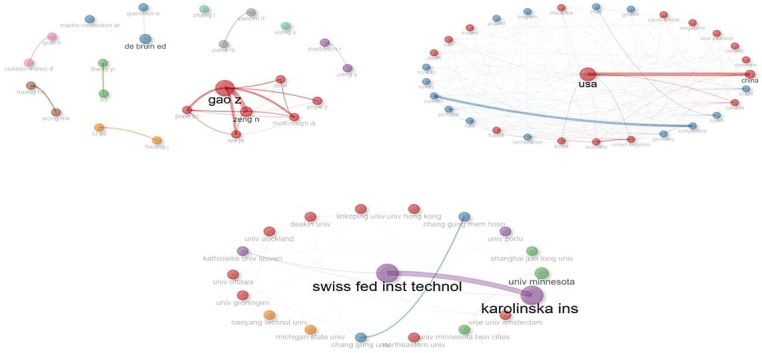
Collaborative network of researchers, countries, and institutions on exergaming.

Figure 6 shows the most productive countries, the most productive authors, the most productive institutions, and the connections between them in exergaming research. One of the largest nodes on the graph, Gao Zan, is seen to collaborate closely with Zeng Nan, indicating that they have conducted many studies together. In terms of collaborations between countries, the United States is at the center, showing a strong collaboration with China. Leading institutions include the Swiss Federal Institute of Technology (ETH Zurich) and the Karolinska Institute, which have been very active and show strong relationships between them.

### Coword Network

**Figure 7.**
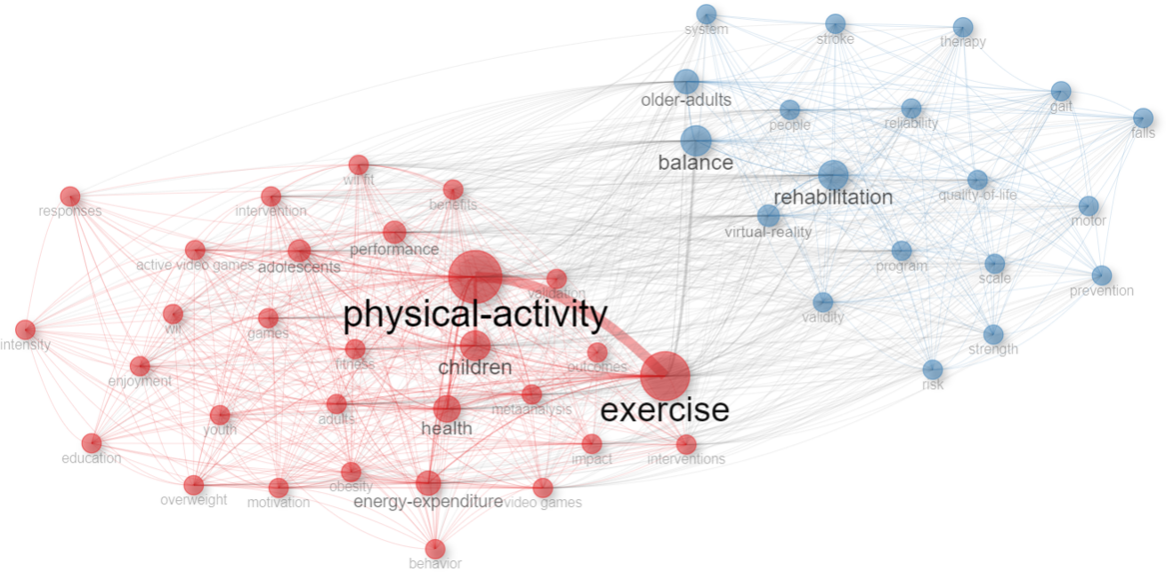
Coword analysis of exergaming studies.

Figure 7 presents a coword analysis based on the frequency of terms used in the titles, keywords, and abstracts of the 1626 included studies, highlighting the most prominent concepts in the field and the relationships between them. Using word cloud analysis in Bibliometrix, the main themes and concepts driving exergaming studies were identified, with PA (n=436), exercise (n=383), rehabilitation (n=218), and balance (n=213) ranking as the most prominent. The high frequency of these terms indicates their central role in exergaming research. Then coword analysis showed that the studies in the field are divided into 2 main clusters. Cluster 1 focuses on PA, exercise, performance, health, and health-related concepts, with these studies primarily targeting children and adolescents. Cluster 2, on the other hand, provides insights into studies focused predominantly on adults and older people, emphasizing rehabilitation, balance, and strength, highlighting the interconnectedness of these concepts.

## Discussion

### Principal Findings

This bibliometric analysis has revealed a significant increase in exergaming research since 2010, peaking during the 2020‐2021 period. Thematic analyses demonstrate that exergaming research is inherently interdisciplinary and spans areas such as health, rehabilitation, and behavior change. Collaboration patterns and trend analyses highlight the potential of exergaming to address broader health challenges, providing critical insights to guide future research directions.

### Annual Scientific Production in Exergaming Research

The annual production of scientific publications on exergaming has shown significant growth since 1997, following the release of the Dance Dance Revolution. A prior bibliometric study focusing on VR, AR, and PA observed a similar growth trend, consistent with our findings [26,43]. A significant increase in publication volume has been observed since 2015, with scientific production peaking in 2020‐2021. This growth reflects advancements in VR and AR technologies as well as increased investments in health-focused gaming applications and the growing role of technology-assisted interventions in health care services [21,44]. Furthermore, this rise can also be attributed to the COVID-19 pandemic, which highlighted the need for home-based solutions to maintain PA, thereby amplifying the significance of exergaming research [45,46]. After 2002, the average citation rate dropped sharply and did not follow a trajectory parallel to the increase in the number of publications. A possible explanation for this is that the growth in the number of studies within the exergaming field led to citations being distributed across a broader range of research. As the field expanded, citations may have been spread among a larger body of work, diminishing the citation impact of earlier studies.

### Journals, Authors, and Papers Featured in Exergaming Research

*Games for Health Journal*, *International Journal of Environmental Research and Public Health*, and *JMIR Serious Games* are leading journals for exergaming research in health and education. These leading journals are essential platforms that facilitate the dissemination of innovative studies, promote interdisciplinary collaboration, and shape the applications of exergaming in improving health and PA outcomes [12,47,48]. According to Bradford’s law, this finding indicates that most of the exergaming research is concentrated in a few core journals, while the remaining publications are distributed across a broader range of sources [49].

When examining the publication performance of the most prolific authors in the field of exergaming, a few notable names stand out, such as Gao Zan and Eling de Bruin. The continued production of relevant research by these authors, along with their numerous publications and high citation frequency, highlights their significant roles in the field of exergaming. According to Lotka’s law, about 60% of researchers are expected to contribute with a single publication, 15% with 2 publications, and 6.67% with 3 publications [50]. Considering Lotka’s law, which reveals the scientific productivity of authors in the field of exergaming, it can be concluded that researchers generally prefer to conduct a single study on this topic, and the number of authors specializing in the field remains limited. This shows that the literature in this field still has room for improvement.

The most cited on exergaming is Biddiss and Irwin’s paper [35], which received 386 citations in total. This paper reviews the potential of exergaming to promote PA among children and adolescents and possibly lays the groundwork for further studies by emphasizing the positive effects of exergaming on child health [35]. Another influential paper is the paper by Ijaz et al [5], which received 345 citations and had the highest annual citation rate of 57.5. This study investigates how VR exergaming increases participants’ motivation and enjoyment [5]. The high citation counts of these papers demonstrate their important contributions to the field of exergaming and their role as key reference points for other researchers.

### Hot Spots in Exergaming Research

In the analysis of trending topics, early studies primarily concentrated on terms such as “obesity,” “childhood,” and “video games,” whereas more specific topics like “virtual reality,” “physical activity,” and “rehabilitation” have gained prominence over time. This shift reflects the integration of advanced technologies and broader health applications into the field [51]. Furthermore, the rising frequency of terms such as “adolescents” and “adults” indicates a widening demographic focus, extending beyond children to include populations of different age groups. This evolution demonstrates the expanding scope of exergaming research, moving from a niche focus on childhood health to a broader application in preventive health care [52], rehabilitation [15], and lifestyle improvement. Finally, terms such as “intention” and “medicine” have more recently started to receive increasing attention, indicating that exergaming is being increasingly explored for behavior change and clinical interventions. Future studies could further enhance its impact by addressing less-explored topics and demographic groups.

Thematic analysis results reveal that fundamental themes, such as “virtual reality,” “randomized controlled trials,” and “physical activity,” constitute the main research topics in exergaming, particularly focusing on health and rehabilitation applications.

Niche themes, including “crossover trials” and “autism spectrum disorders,” delve into specialized research areas, allowing for in-depth studies on specific disease groups and research methods [53]. Themes such as “Wii Fit” and “physical education” efforts for school children represent topics that once gained popularity in exergaming research but have lost attention due to technological advancements and shifting research priorities. Future studies could explore niche themes in greater depth to discover new application areas and diversify basic themes to expand the potential of exergaming to broader audiences.

### Author, Country, and Institutional Collaborations in Exergaming Research

Authors such as Gao Zan and Zeng Nan are notable for their extensive collaborations, which may stem from their shared interest in studying the effects of exergaming on health and PA. Furthermore, 26.88% (n=437.07) of the 1626 studies in the field of exergaming had international author collaborations, indicating that more than 1 researcher worked together.

The United States stands out as the most productive country, maintaining strong collaborations with many other nations. China also plays a crucial role, collaborating closely with the United States. These collaborations have facilitated extensive research on exergaming, supported by substantial scientific research funds, robust research infrastructures, and leading technological advancements. Our findings are consistent with bibliometric studies focusing on technologies like VR and AR, commonly used in exergaming applications [26,27].

Leading institutions like the Swiss Federal Institute of Technology (ETH Zurich) and Karolinska Institute are at the forefront of this field. ETH Zurich, recognized for its expertise in technology and engineering, contributes considerably to the development and application of exergaming devices [54], while Karolinska Institute is prominent in medical research and clinical applications [55]. These institutional collaborations result in extensive and impactful studies investigating various applications of exergaming in fields such as health care and rehabilitation.

### Future Research Suggestions

First, previous literature has shown that group-based exergames enhance motivation, promote sustained play, and improve self-efficacy. Family-friendly games like Ring Fit Adventure have the potential to increase PA while strengthening parent-child bonds. Future research should investigate the impact of these games on family dynamics and address critical issues such as “adherence,” “sustainability,” and “transfer effects.” Second, significant increases in energy expenditure as a result of exergaming might be of little importance if one compensates by increasing energy intake. Therefore, promoting exergaming as an obesity prevention tool also requires the quantification of energy intake to adequately assess overall energy balance. Without considering both sides of the equation, energy expenditure and intake, it is difficult to determine the true effectiveness of exergaming in managing or preventing obesity. Future studies should incorporate both metrics to provide a clearer understanding of exergaming’s potential health benefits. Third, exergaming has not been widely used in young children such as preschoolers; thus, it needs more research in this population. Fourth, with the advancement of technology and the introduction of new active video games, it is important to explore how these new games can promote PA and other health-related outcomes. Fifth, research could examine how exergaming can be integrated with technological advances and the potential benefits of this integration in health and education. Sixth, more research is needed on the social impacts and community-based applications of exergaming. Finally, further research on the economic issues and financial sustainability of exergaming is critical to assess its cost-effectiveness, economic benefits, and investment opportunities.

### Limitations

This study has various limitations. First, only the WoS database was used because the robust analytical capabilities of the Bibliometrix R package with WoS data enhanced the reliability of our results, and WoS’s “Keyword Plus” feature identified contextually significant keywords, thereby broadening the scope of the study. However, relying on a single database may exclude relevant sources from other databases, which is considered a limitation. Second, the R-based Bibliometrix tool was used for the analysis and visualization processes in this study, as it provided a sufficient platform aligned with the study’s scope without incurring additional software costs. However, the exclusion of additional features offered by alternative software is considered a limitation of this study. Third, the metrics and indicators used, such as citation counts, journal impact factors, and authors’ h-indexes, may not fully capture the quality of the research and potentially overestimate the impact of particular studies [56]. Finally, bibliometric methods provide an overview of a research field; however, this study does not offer an in-depth analysis of influential papers and authors. To gain a more comprehensive understanding of research impact and trends, bibliometric analyses should be complemented with other evaluation methods.

### Conclusions

This study highlights the growing importance of exergaming research as a key indicator of the increasing integration of technology into the health domain. The COVID-19 pandemic has especially highlighted the value and relevance of home-based solutions in this field. The interdisciplinary nature of exergaming offers significant potential for the development of innovative approaches by bringing together technology and health applications. The thematic evolution of research demonstrates that exergaming has progressed beyond being merely a tool for children’s entertainment or PA promotion, expanding into broader health contexts such as rehabilitation, behavior modification, and applications for diverse age groups. This trend suggests that exergaming could play a more active role in public health policies in the future. Future research focusing on underexplored demographic groups, emerging technologies, and niche health applications is likely to further enhance the impact of exergaming.

## Supplementary material

10.2196/66738 Multimedia Appendix 1 Search strategy protocol.

10.2196/66738 Multimedia Appendix 2 Data from all included studies.

10.2196/66738 Multimedia Appendix 3 Local impact of the top journals on exergaming.

10.2196/66738 Multimedia Appendix 4 Scientific production of journals according to Bradford’s law.

10.2196/66738 Multimedia Appendix 5 Author productivity findings according to Lotka’s law.

## Abbreviations

AR: augmented reality
PA: physical activity
RQ: research question
TC: total citation
VR: virtual reality
WoS: Web of Science
