# Supplementary material for: Mapping Hot Spots and Global Research Trends in Exergaming Between 1997 and 2024: Bibliometric Analysis
Source: Interact J Med Res. 2025 Aug 25;14:e66738. doi: 10.2196/66738 (PMC12377698; doi:10.2196/66738)
Supplement: Multimedia Appendix 1 [file ijmr-v14-e66738-s001.docx]

**WoS**

***Advanced Search***

(TI=(“exergame*” OR “gamercising” OR “exergaming” OR “gamercize*” OR “active video game*” OR “active video gaming” OR “exertion game*” OR “exertion gaming” OR “fitness game*” OR “exercis* game*” OR “fitness video game*” OR “exercis* video game*” OR “activity promoting video game*” OR interactive video game* and physical activit* OR interactive video game* and exercis* OR virtual reality and exercis* OR augmented reality and exercis* OR virtual reality and physical activit* OR augmented reality and physical activit* OR serious game* and physical activit* OR serious game* and exercis*))
