# Supplementary material for: Mapping Hot Spots and Global Research Trends in Exergaming Between 1997 and 2024: Bibliometric Analysis
Source: Interact J Med Res. 2025 Aug 25;14:e66738. doi: 10.2196/66738 (PMC12377698; doi:10.2196/66738)
Supplement: Multimedia Appendix 3 [file ijmr-v14-e66738-s003.pdf]

Element	h_ index	g_ index	m_ index	TC[a]	NP[b]	PY start[c]
“Games For Health Journal”	30	44	2,143	2651	139	2012
“International Journal of Environmental Research and Public Health”	19	30	1,727	1071	54	2015
“JMIR Serious Games”	16	24	1,455	775	63	2015
“Journal of Neuroengineering and Rehabilitation”	16	23	1,143	967	23	2012
“Cyberpsychology Behavior And Social Networking”	14	15	0,875	858	15	2010
“Journal of Clinical Medicine”	14	27	1,75	756	28	2018
“Journal of Medical Internet Research”	14	22	1,077	696	22	2013
“Plos One”	14	25	1,077	666	32	2013
“Archives of Physical Medicine And Rehabilitation”	12	14	0,444	968	14	1999
“Computers in Human Behavior”	12	16	0,522	637	16	2003

[a]TC: total citiations
[b]NP: number of publications
[c]PY Start: publication year start
