# Supplementary figures and images for: Mapping Hot Spots and Global Research Trends in Exergaming Between 1997 and 2024: Bibliometric Analysis
Source: Interact J Med Res. 2025 Aug 25;14:e66738. doi: 10.2196/66738 (PMC12377698; doi:10.2196/66738)

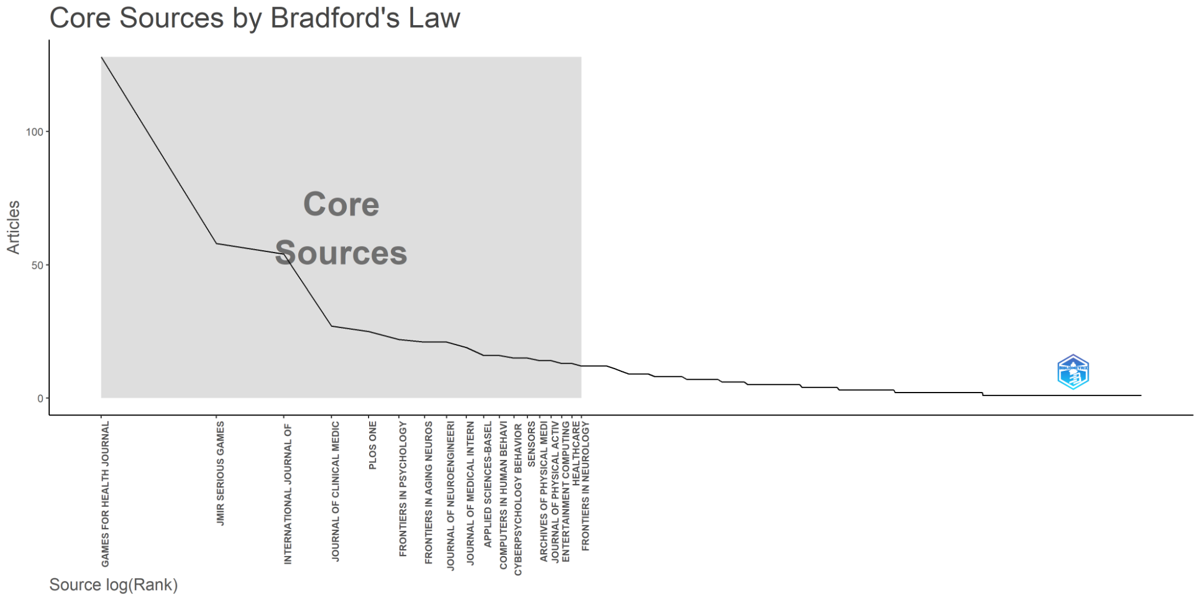

Supplement: Multimedia Appendix 4 [file ijmr-v14-e66738-s004.png]

| Documents written | Authors, n | Proportion of Authors |
|-------------------|------------|-----------------------|
| 1                 | 5226       | 0,797                 |
| 2                 | 842        | 0,128                 |
| 3                 | 245        | 0,037                 |
| 4                 | 105        | 0,016                 |
| 5                 | 50         | 0,008                 |
| 6                 | 29         | 0,004                 |
| 7                 | 22         | 0,003                 |
| 8                 | 16         | 0,002                 |
| 9                 | 3          | 0                     |
| 10                | 3          | 0                     |

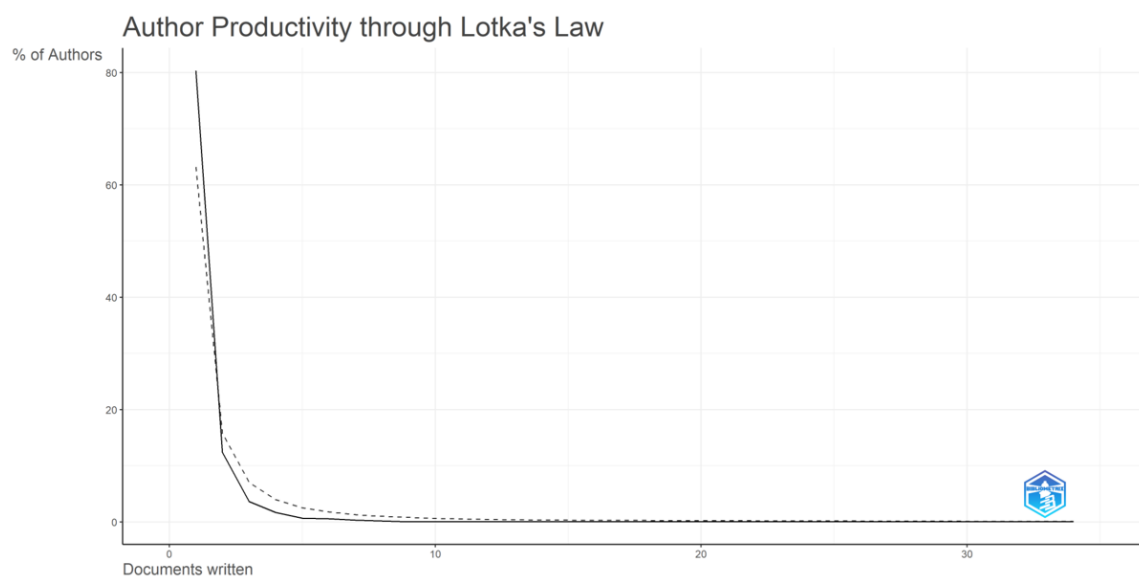

Supplement: Multimedia Appendix 5 [file ijmr-v14-e66738-s005.pdf]
